# Adipokines are Associated With Hypertension in Metabolically Healthy Obese (MHO) Children and Adolescents: A Prospective Population-Based Cohort Study

**DOI:** 10.2188/jea.JE20160141

**Published:** 2018-01-05

**Authors:** Wenqing Ding, Hong Cheng, Fangfang Chen, Yinkun Yan, Meixian Zhang, Xiaoyuan Zhao, Dongqing Hou, Jie Mi

**Affiliations:** 1 Department of Epidemiology, Capital Institute of Pediatrics, Beijing, China; 2 Department of Children and Adolescents Health Care, School of Public Health, Ningxia Medical University, Yinchuan, China

**Keywords:** adipokines, hypertension, obesity, metabolic

## Abstract

**Background:**

The potential mechanism underlying the relationship between the risk of cardiovascular diseases and metabolically healthy obese (MHO) individuals remains unclear. The aim of the study was to prospectively investigate the potential role of the adipokines in the association between the MHO phenotype and hypertension in children and adolescents.

**Methods:**

A total of 1184 participants at baseline were recruited from a cohort of the Beijing Child and Adolescent Metabolic Syndrome (BCAMS) study. The participants were classified according to their body mass index (BMI) and metabolic syndrome (MS) components. The levels of the adipokines, including leptin, adiponectin, and resistin, were measured.

**Results:**

MHO individuals had higher leptin levels (11.58 ug/L vs 1.20 ug/L), leptin/adiponectin ratio (1.18 vs 0.07), and lower adiponectin (11.65 ug/L vs 15.64 ug/L) levels compared to metabolically healthy normal-weight individuals (all *P* < 0.05). Compared to metabolically healthy normal-weight individuals, the prevalence of high leptin levels (26.5% vs 0.4%), low adiponectin levels (17.9% vs 6.3%) and a high leptin/adiponectin ratio (26.0% vs 2.1%) was higher in MHO individuals (all *P* < 0.01). The MHO individuals with abnormal adipokines were significantly more likely to developing hypertension (high leptin, relative risk 11.04; 95% confidence interval, 1.18–103.35; and high leptin/adiponectin ratio, relative risk 9.88; 95% confidence interval, 1.11–87.97) compared to metabolically healthy normal-weight individuals with normal adipokine levels.

**Conclusions:**

The abnormal adipokine levels contribute to the increased hypertension risk in MHO children and adolescents. The non-traditional risk factors should be highlighted in MHO children and adolescents in clinical practice and research.

## INTRODUCTION

The prevalence of obesity and type 2 diabetes has rapidly increased in Asian countries as a result of the Westernization of lifestyle, and the rates of increase do not show signs of slowing over the past few decades.^1^ Metabolic syndrome is also growing into a significant public health problem. Particularly vulnerable populations include children and adolescents. Moreover, studies indicate that South and East Asians have higher proportion of body fat, prominent abdominal obesity, and greater metabolic responses to obesity compared with their western counterparts with similar body mass index (BMI) values.^2^^,^^3^ Other studies show that South Asian people have more severe atherogenic dyslipidemia, endothelial dysfunction, and glucose intolerance than white Caucasians, even at an early age.^4^ With rapid economic and accelerated pace of nutrition transition changes in China, general and abdominal obesity in Chinese children and adolescents also increased significantly over the past few decades.^5^ Thus, it is very important to investigate obesity and related metabolic dysfunction among Asian people, especially for children and adolescents.

Recent research has focused on the novel obesity phenotype of metabolically healthy obese (MHO), which is characterized as having favorable cardio-metabolic risk factors despite a high BMI and fat mass. MHO individuals are different from metabolically unhealthy obese (MUO) individuals with regards to the distribution of body composition, the inflammation profile,^6^ and cardiovascular disease (CVD) outcomes.^7^ Therefore, distinguishing MHO from MUO individuals is of public health and clinic significance, and we must direct the limited resources available for prevention of obesity-associated metabolic complications to the individuals who are at highest risk.^8^

However, recent studies have revealed inconsistent results regarding the relationship between the MHO phenotype and CVD. Evidence from a longitudinal study has shown that MHO participants were not at increased risk of CVD over a period of 7 years compared to the metabolically healthy normal-weight individuals.^9^ However, a more recent systematic review and meta-analysis study in adult populations showed that MHO individuals were at increased risk for adverse long-term outcomes compared to metabolically healthy normal-weight individuals. Evidence from our previous study also demonstrated that MHO children and adolescents still had an elevated incidence of hypertension, irrespective of metabolic abnormalities as defined by metabolic syndrome (MS) or insulin resistance.^10^ Therefore, the MHO phenotype should not be regarded as a healthy pattern of increased weight.^11^ Taken together, the current CVD metabolic risk factors, such as MS or insulin resistance, might not be sufficient to demonstrate the complete range of metabolic abnormalities in MHO individuals, and additional metabolic risk factors might explain the positive relationship between MHO and CVD outcomes.^12^

Leptin, adiponectin, and the leptin:adiponectin ratio have been shown to be informative biomarkers for obesity, MS, Type 2 diabetes, and CVD in children and adolescents.^13^^,^^14^ Leptin and adiponectin have been recommended as adipose tissue biomarkers in defining of MS for research purposes.^15^ In recent years, the leptin:adiponectin ratio has been suggested to deserve further consideration and should be used as a possible component of MS.^16^ However, it is unknown whether the use of these adipokines as additional components of MS are effective indicators for an increased CVD risk in MHO individuals, given that the MHO phenotype begins in childhood and continues into adulthood.^17^ Therefore, identifying MHO from childhood might become increasingly important to stratify individuals for public health prevention and for the clinical treatment of obesity to decrease the prevalence of obesity-related chronic diseases in adulthood. The potential mechanism explaining the relationship of the MHO phenotype and cardiovascular risk has been described in adults,^18^^,^^19^ but limited data were shown in children and adolescents, especially data concerning the role of adipokines. Hence, the aim of the study is to determine the potential role of adipokines in the association between the MHO phenotype and hypertension in children and adolescents.

## METHODS

### Study participants

Subjects were recruited from a cross-sectional population-based survey from the Beijing Child and Adolescent Metabolic Syndrome (BCAMS) study. The study conducted a stratified, randomly clustered sampling design to select subjects from resident communities. Three out of the ten suburb and rural districts and four out of the eight urban districts were randomly selected. A total of 13 kindergartens, 22 primary schools, and 7 high schools were chosen for different age subgroups. Records of 19,593 children aged 6–18 years who had complete information of anthropometric measurement were recruited in 2004. Of them, a total of 2,661 children aged 6–14 years (55% boys, *n* = 1,463; 45% girls, *n* = 1,198) who completed the further venipuncture blood samples test with valid data on variables needed for defining MS at baseline and were still at school after a 6-year interval were examined again. For the longitudinal analysis, 472 individuals who were examined and found to be hypertension at baseline and 1,005 individuals who did not attend the follow-up examinations were excluded. The remaining 1,184 participants who had complete systolic and diastolic blood pressure (SBP and DBP, respectively) measurements were eligible for the analysis.

Signed informed consent was obtained from all participants and/or their parents or guardians. The BCAMS protocol was approved by the Ethics Committee at the Capital Institute of Pediatrics in Beijing.

### Baseline and follow-up investigations

A comprehensive health examination, which was completed by all participants, included the evaluation of anthropometrics indices and the assessment of biological specimens. Information about demographic information, cigarette consumption, and physical activity were also provided by the participants, as well as information about dietary habits using a food frequency questionnaire (FFQ) and a family history of hypertension. All of the examinations were administered by trained health professionals according to a standardized protocol. Body mass index (BMI) was calculated using the following formula: weight/height^2^ (kg/m^2^). Fat mass percentage (FMP) was measured using a body composition analyzer (TBF-300A Body Composition Analyzer; Tanita, Tokyo, Japan). Pubertal development was assessed according to the Tanner stages of breast development in girls and testicular volume in boys.^20^

Venous blood samples were collected after an at least a 12-hour overnight fast. Fasting plasma glucose (glucose oxidase method) and serum lipids (enzymatic methods) were directly measured using the 7,060 chemistry analyzer (Hitachi, Tokyo, Japan). The high-density lipoprotein (HDL) cholesterol and low-density lipoprotein (LDL) cholesterol levels were measured directly. Serum leptin levels were measured using enzyme-linked immunosorbent assays (ELISA) with rabbit polyclonal (PAb) and mouse monoclonal (MAb) antihuman leptin antibodies, prepared following the injection of human leptin (R&D System, Minneapolis, MN, USA).^21^ Serum adiponectin levels were measured using ELISA. The intra- and inter-assay coefficients of variation were <5.4% and <8.5%, respectively. The leptin:adiponectin (L:A) ratio was also calculated in the study. Serum resistin levels were measured using the ELISA kit (Phoenix Pharmaceuticals Inc., Belmont, CA, USA), with intra- and inter-assay coefficients of variation of <10% and <5%, respectively.

The follow-up examination was conducted in 2010. The collection of sociodemographic characteristics and anthropometric measurements, including height, weight, waist circumference (WC), and blood pressure were taken according to the standard protocol that was used during the baseline examination.

### Definitions

The International Obesity Task Force (IOTF) reference was used to classify normal weight, overweight, and obesity.^22^ Metabolic abnormalities were defined as the presence of two or more of the following five components of MS^23^: 1) central obesity, defined as WC ≥90th percentile for age and sex; 2) elevated SBP and/or DBP, defined as ≥90th percentile for age and sex; 3) hypertriglyceridemia, defined as triglyceride (TG) levels ≥1.24 mmol/L; 4) low serum HDL cholesterol levels, defined as ≤1.03 mmol/L; and 5) impaired fasting glucose, defined as ≥5.6 mmol/L. Thus, we defined MHO as obesity with <2 MS components and MUO as obesity with ≥2 MS components. Abnormal adipokines were defined as adipokines levels ≥85th percentile for age and sex, with the exception of low adiponectin, which was defined as adiponectin levels ≤15th percentile for age and sex.

### Blood pressure measurement and hypertension incidence

During the baseline and follow-up visits, BP was measured by trained examiners according to a standardized protocol with the individuals in the sitting position using an appropriately-sized cuff and a mercury sphygmomanometer.^24^ After a rest period of at least 15 minutes, BP measurements were repeated three times after 1–2 minute intervals, and the average values of the last two measurements were used for the analysis. The first and fourth phases of the Korotkoff sounds were used to for the systolic BP (SBP) and the diastolic BP (DBP), respectively. An individual was noted as having hypertension at baseline if the SBP and (or) DBP were greater than or equal to the 95^th^ percentile for age and sex.^25^ The same criteria were applied to define the incidence hypertension at the follow-up examination.

### Statistical analyses

The data are expressed as the mean and standard deviation (SD), median and 25^th^–75^th^ percentiles, or as proportions. Skewed distributions were logarithmically transformed for analysis. Differences between the categories of BMI and metabolic status were determined using analysis of variance and a Bonferroni post-hoc comparison test for continuous variables or the chi-squared test for categorical variables. Adipokines levels were compared among subjects by BMI and metabolic status at baseline using analysis of covariance, and the prevalence of abnormal adipokines was determined using multivariate logistic regression analysis with adjustment for age, sex, and pubertal stage. Pearson’s correlation test was used for correlation analyses. We used multivariate logistic regression models to estimate relative risks (RRs) and 95% confidence intervals (CIs) for incident hypertension on abnormal adipokines and combined MHO phenotypes with abnormal adipokines. These models were adjusted for confounders, including age, sex, BMI, fathers’ BMI, mothers’ BMI, pubertal stage, physical activity, and family history of hypertension. All analyses were conducted using SPSS, version 13.0 (SPSS Inc., Chicago, IL, USA), with statistical significance set at *P* < 0.05.

## RESULTS

### Baseline characteristics

The subjects (*n* = 1,184) were an average age of 10.0 (SD, 2.1) years, and 45.7% (*n* = 541) were girls. The demographics and clinical characteristics of the subjects classified by BMI and metabolic status at baseline are shown in Table 1. Compared to the metabolically healthy normal-weight individuals, the MHO individuals had higher BMI, WC, FMP, SBP, TG, and LDL cholesterol levels, and lower HDL cholesterol levels (all *P* < 0.05). MUO individuals had higher BMI, WC, FMP, blood pressure, TG, and fasting glucose levels but lower levels of HDL cholesterol compared to the MHO individuals (all *P* < 0.05). The levels of physical activity, frequent dietary consumption (including the consumption of fruits and vegetables as well as the consumption of high fat/sweetened foods), and annual family income were not significantly different between the MHO and the metabolically healthy normal-weight individuals (Table 1).

**Table 1.** Demographics and clinical characteristics of subjects according to BMI and metabolic status at baseline

Characteristics	Metabolically healthy			Metabolically unhealthy			*P*-value
	
	Normal weight	Overweight	Obese (MHO)	Normal weight	Overweight	Obese	
*n*	517	197	130	43	208	334	
Age (years)	9.7(2.16)	9.7(1.86)	9.2(1.87)^*^	10.5(2.11)^*^	10.8(2.13)^*^	10.1(2.09)^*^^Δ^	<0.001
Sex (boys, %)	48.2	48.7	72.3^*^	44.2	41.8	67.7^*^	<0.001
Pubertal stage (prepuberty, %)^a^	70.1	63.6^*^	71.9^*^	18.2	56.9	73.5	0.001
BMI (kg m^−2^)	16.5(1.98)	21.8(1.97)^*^	25.2(2.54)^*^	18.13(2.52)^*^	21.8(1.97)^*^	27.7(3.06)^*^^Δ^	<0.001
WC (cm)	57.9(5.74)	70.3(7.34)^*^	78.9(8.92)^*^	63.4(8.19)^*^	76.1(7.21)^*^	83.4(8.71)^*^^Δ^	<0.001
FMP (%)	16.0(4.59)	25.7(5.12)^*^	30.8(6.51)^*^	20.6(6.12)^*^	29.2(5.55)^*^	32.6(6.84)^*^^Δ^	<0.001
SBP (mm Hg)	98(11)	104(9)^*^	108(9)^*^	108(11)^*^	111(10)^*^	114(10)^*^^Δ^	<0.001
DBP (mm Hg)	63(8)	66(7)^*^	68(7)	71(9)^*^	70(8)^*^	73(9)^*^^Δ^	<0.001
TG (mmol l^−1^)^b^	0.76(0.59,0.96)	0.88(0.65,1.06)^*^	0.89(0.68,1.12)^*^	1.46(1.10,1.70)^*^	1.28(0.90,1.62)^*^	1.30(0.92,1.71)^*^^Δ^	<0.001
HDL-C (mmol l^−1^)	1.61(0.31)	1.46(0.27)^*^	1.39(0.24)^*^	1.47(0.42)^*^	1.31(0.29)^*^	1.31(0.28)^*^^Δ^	<0.001
LDL-C (mmol l^−1^)	2.32(0.74)	2.47(0.64)^*^	2.52(0.51)^*^	2.19(0.56)	2.46(0.60)^*^	2.58(0.60)^*^	<0.001
TC (mmol l^−1^)	4.12(0.79)	4.11(0.69)	4.11(0.57)	4.19(0.75)	4.13(0.69)	4.25(0.71)	NS
Fasting glucose (mmol l^−1^)	5.25(0.54)	5.19(0.42)	5.08(0.37)^*^	5.67(0.59)^*^	5.64(1.15)^*^	5.56(0.66)^*^^Δ^	<0.001
Physical activity (%)^c^	87.7	92.1	89.6	86.1	81.6	85.4	NS
Frequent dietary consumption							
Fruits and vegetables (every day)	58.9	56.5	51.2	56.1	56.9	52.7	NS
High fat foods/sweetened (≥1 times/per 2 weeks)^d^	40.4	76.5^*^	99.2^*^	100.0	99.5^*^	98.2^*^	<0.001
Family history of hypertension (%)^e^	18.2	23.9	32.3^*^	14.0	16.3	20.1	0.003
Annual family income (ten thousand Yuan)^b^	2.4(1.2,6.0)	3.0(1.5,5.0)	3.0(2.0,4.8)	1.8(0.9,3.1)	3.0(1.8,6.0)	2.4(1.6,4.0)	NS
Fathers’ BMI	21.2(3.16)	21.5(2.67)	22.4(31.3)^*^	21.2(3.16)	22.1(2.68)^*^	22.2(2.96)^*^	0.001
Mother’s BMI	17.9 (2.45)	19.0(2.57)^*^	19.7(3.27)^*^	17.9(1.91)	19.1(2.41)^*^	19.6(2.75)^*^	<0.001

BMI, body mass index; DBP, diastolic blood pressure; FMP, fat mass percentage; HDL-C, high-density lipoprotein cholesterol; LDL-C, low-density lipoprotein cholesterol; MHO, metabolically healthy obesity; NS, not significant; SBP, systolic blood pressure; TC, total cholesterol; TG, triglyceride; WC, waist circumference. Normally distributed data are expressed as mean ± s.d. The median and interquartile range was used for skewed variables. ^*^: *P* < 0.05 for post hoc pairwise comparisons with metabolically healthy normal weights; ^Δ^: *P* < 0.05 for comparisons between MHO and MUO. ^a^: Prepuberty: Tanner stage 1. ^b^: Skewed distributions were logarithmically transformed for statistical tests. ^c^: Over one time biweekly and over 30 min per time. ^d^: High fat food: consumption of at least one meat, fried food or western fast food. ^e^: Presence of at least one parent with hypertension.

In view of the influence of loss to follow-up, the comparison between characteristics of followed and non-followed subjects at baseline were also analyzed (eTable 1). The followed subjects had higher BMI, WC, FMP, and blood pressure but lower levels of HDL cholesterol compared to non-followed subjects (all *P* < 0.05). Subjects who attended follow-up were more likely in prepuberty. There were no statistically or clinically significant difference in age, sex, family history of hypertension, TG levels, LDL cholesterol levels, total cholesterol levels, and annual family income between followed and non-followed subjects (all *P* > 0.05).

### Comparison of adipokine levels and the prevalence of abnormal adipokines in subjects classified by BMI and metabolic status at baseline

The levels of adipokines in subjects classified by BMI and metabolic status at baseline are shown in Table 2. After adjusting for sex, age, and pubertal stage, the MHO individuals had higher leptin levels, lower adiponectin levels, and a higher L:A ratio compared to metabolically healthy normal-weight individuals. MUO individuals had worse adipokines profiles, including lower adiponectin levels, and a higher leptin/adiponectin ratio compared to the MHO individuals.

**Table 2.** Levels of adipokines among subjects according to BMI and metabolic status at baseline

	Metabolically healthy			Metabolically unhealthy			*P*-value
	
	Normal weight	Overweight	Obese (MHO)	Normal weight	Overweight	Obese	
Serum leptin, ug l^−1 a^	1.20(0.57,2.43)	7.71(4.73,13.84)^*^	11.58(7.73,20.86)^*^	3.25(2.25,13.44)^*^	10.15(4.63,16.83)^*^	15.08(8.24,27.10)^*^	<0.001
Resistin, ug l^−1 a^	14.38(10.36,21.86)	15.34(10.90,21.36)	17.13(11.69,27.08)	20.03(15.42,26.78)	16.38(10.74,22.96)	16.43(11.53,24.46)	NS
Adiponectin, mg l^−1 a^	15.64(11.06,20.89)	12.96(9.94,16.42)^*^	11.65(7.73,16.12)^*^	13.80(8.10,24.90)^*^	9.66(6.57,14.28)^*^	9.09(5.63,12.89)^*^^Δ^	<0.001
Leptin:adiponectin ratio^a^	0.07(0.03,0.18)	0.58(0.35,1.08)^*^	1.18(0.49,2.06)^*^	0.44(0.14,1.16)^*^	0.78(0.54,1.99)^*^	1.47(0.77,2.75)^*^^Δ^	<0.001

MHO, metabolically healthy obesity; NS, not significant.

^a^Normally distributed data are expressed as mean ± s.d. The median and interquartile range were used for skewed variables.

^*^P < 0.05 for post hoc pairwise comparisons with metabolically healthy normal weights.

^Δ^*P* < 0.05 for comparisons between MHO and MUO.

**Figure 1.**
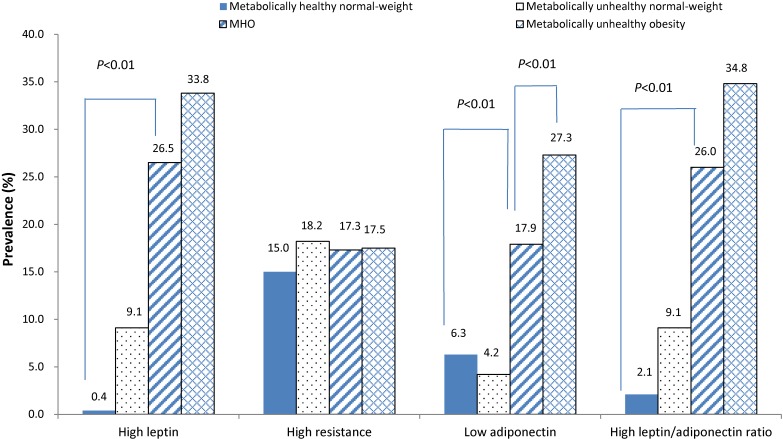
Prevalence of abnormal adipokines among subjects by BMI and metabolic status at baseline. Abnormal adipokines were defined as adipokines levels ≥85th percentile for age and sex, with the exception of low adiponectin, which was defined as adiponectin levels ≤15th percentile for age and sex. Metabolic abnormalities were defined by the presence of two or more of the five components of MS. Obesity was defined based on the International Obesity Task Force (IOTF). The prevalence of high leptin, lower adiponectin and high leptin/adiponectin ratio in MHO individuals were significantly higher than in metabolically healthy normal-weight individuals (all *P* < 0.01). MHO, metabolically healthy obesity.

Similarly, the prevalence of high leptin levels, low adiponectin levels and a high leptin/adiponectin ratio in MHO individuals was significantly higher than in metabolically healthy normal-weight individuals. Compared to MHO individuals, MUO individuals had a higher prevalence of lower adiponectin levels (*P* < 0.01) (Figure 1).

### Correlations between the adipokines and blood pressure

**Figure 2.**
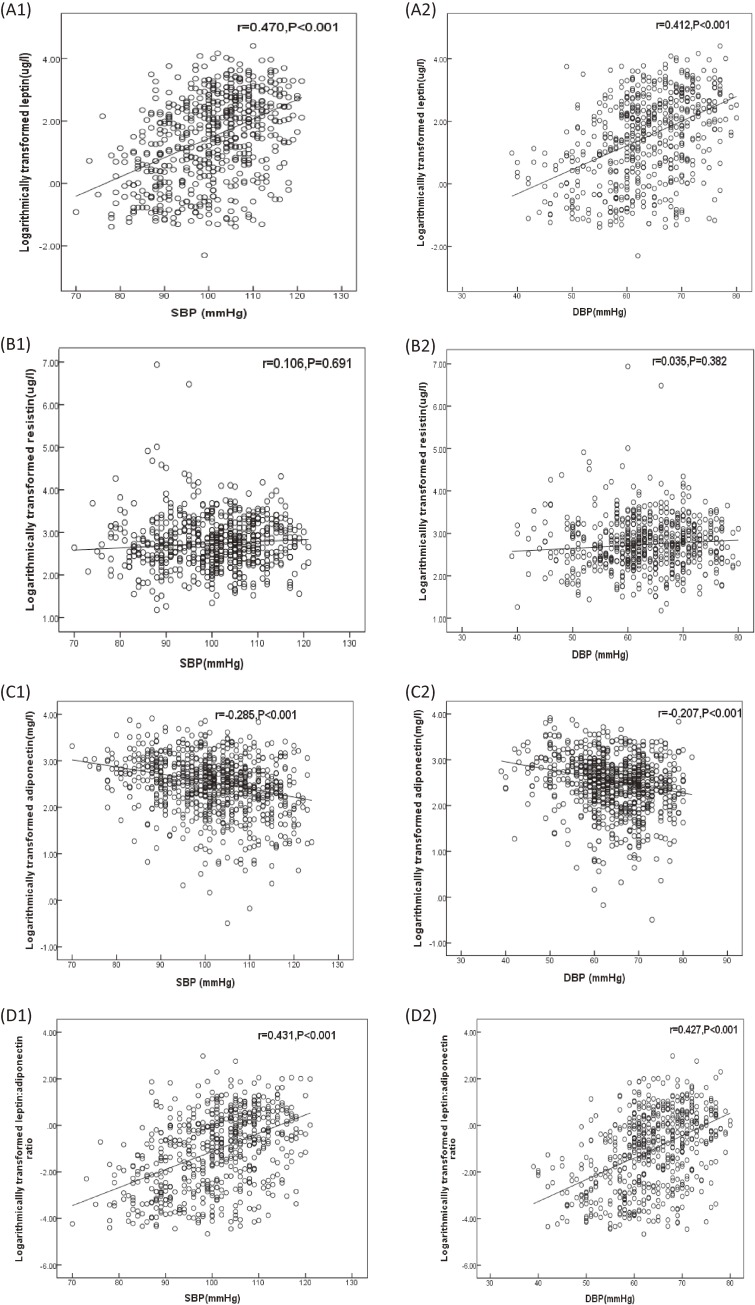
Correlation analysis between logarithmically transformed adipokines and systolic blood pressure (SBP) and diastolic blood pressure (DBP) among children and adolescents at baseline. A1, Correlation between logarithmically transformed leptin and SBP; A2, Correlation between logarithmically transformed leptin and SBP; B1, Correlation between logarithmically transformed resistin and SBP; B2, Correlation between logarithmically transformed resistin and DBP; C1, Correlation between logarithmically transformed adiponectin and SBP; C2, Correlation between logarithmically transformed adiponectin and DBP; D1, Correlation between logarithmically transformed leptin: adiponectin ratio and SBP; and D2, Correlation between logarithmically transformed leptin: adiponectin ratio and DBP.

Correlation analysis for the adipokines and blood pressure indicated that the leptin levels were positively associated with SBP and DBP (*r* = 0.470, *P* < 0.001; and *r* = 0.412, *P* < 0.001, respectively). The adiponectin levels were inversely correctly with SBP and DBP (*r* = −0.285, *P* < 0.001; and *r* = −0.207, *P* < 0.001, respectively). Furthermore, the ratios of leptin/adiponectin were positively associated with SBP and DBP (*r* = 0.431, *P* < 0.001; and *r* = 0.427, *P* < 0.001, respectively), but the resistin levels were not associated with SBP and DBP, respectively (Figure 2).

### Associations between hypertension and different combinations of MHO phenotype and abnormal adipokines

Longitudinal risk of incident hypertension in relation to baseline abnormal adipokines levels are reported in Table 3. Compared with the normal adipokines levels, individuals with high leptin levels and a high L:A ratio demonstrated an increased risk of developing hypertension (RR 2.10; 95% CI, 1.19–3.73 and RR 2.19; 95% CI, 1.23–3.90, respectively), after adjustment for confounders.

**Table 3.** Relative risks for incident hypertension in relation to baseline leptin, adiponectin, and leptin:adiponectin ratio

Adipokines	Number of hypertension cases /Number of at risk	Model 1		Model 2	
	
		RR	95% CI	RR	95% CI
Leptin, ug l^−1^					
Normal	99/524	1 (reference)		1 (reference)	
High	35/77	3.57	2.17–5.89	2.10	1.19–3.73
Adiponectin, mg l^−1^					
Normal	141/700	1 (reference)		1 (reference)	
Low	36/125	1.61	1.04–2.46	0.96	0.52–1.82
High leptin/adiponectin ratio					
Normal	98/519	1 (reference)		1 (reference)	
High	35/79	3.42	2.08–5.61	2.19	1.23–3.90

CI, confidence interval; RR, relative risk.

Model 1: unadjusted for confounding factors.

Model 2: Adjusted for age, sex, BMI, fathers’ BMI, mothers’ BMI, pubertal stage, physical activity and family history of hypertension.

Table 4 shows the multivariable-adjusted RRs for hypertension incidence according to different combinations of MHO phenotypes and abnormal adipokines. Compared with metabolically healthy normal-weight individuals with normal adipokines levels, the MHO individuals with normal adipokines levels demonstrated a significantly increased risk of developing hypertension, after adjusting for confounders (model 2). In contrast, the MHO individuals with high leptin levels and high L:A ratio were significantly more likely to developing hypertension (RR 11.04; 95% CI, 1.18–103.35 and RR 9.88; 95% CI, 1.11–87.97, respectively).

**Table 4.** Relative risks for incident hypertension according to different combinations of MHO phenotypes and abnormal adipokines

	Adipokines	Number of hypertension cases /Number of at risk	Model 1		Model 2	
	
			RR	95% CI	RR	95% CI
MHO	Low adiponectin					
−	−	31/332	1 (reference)		1 (reference)	
−	+	2/24	0.88	0.19–3.93	0.58	0.07–7.84
+	−	32/72	7.77	4.29–14.07	6.14	1.02–36.87
+	+	4/16	3.24	0.98–10.64	2.75	0.25–30.54
MHO	High leptin					
−	−	25/256	1 (reference)		1 (reference)	
−	+	0/1	—	—	—	—
+	−	24/57	6.72	3.44–13.11	6.54	1.01–40.21
+	+	9/20	7.56	2.85–19.99	11.04	1.18–103.35
MHO	High leptin/adiponectin ratio					
−	−	24/251	1 (reference)		1 (reference)	
−	+	1/6	1.89	0.21–16.87	2.82	0.27–29.67
+	−	25/56	7.63	3.88–14.97	7.96	1.28–49.39
+	+	8/19	6.88	2.52–18.76	9.88	1.11–87.97

CI, confidence interval; MHO, metabolically healthy obesity; RR, relative risk.

Model 1: unadjusted for confounding factors.

Model 2: Adjusted for age, sex, BMI, fathers’ BMI, mothers’ BMI, pubertal stage, physical activity and family history of hypertension.

## DISCUSSION

In this cohort study, we observed a significant and positive association between abnormal adipokines and an increased hypertension risk among MHO individuals in children and adolescents in China.

Whether the MHO individuals have an elevated risk of CVD is still controversial. Studies from Korea and Taiwan suggested that the MHO phenotypes were associated with the development of hypertension in adulthood.^26^^,^^27^ However, a recent study revealed that MHO individuals did not carry excess risk of CVD mortality when compared with metabolically healthy normal-weight individuals using a homeostasis model assessment index to assess metabolic status.^28^ The different results among the previous studies might be related to different definitions of metabolic health.^28^ In our previous study, the MHO phenotype, which was defined as obesity with MS components or insulin resistance, were not benign conditions and still carried an elevated risk of hypertension among children and adolescents in China.^10^

The mechanism that links the MHO phenotype to an increased risk of hypertension remains unclear. Leptin, which was the first adipokine discovered and is secreted by adipose tissue, plays a major role in the control of body fat stores through the coordinated regulation of feeding behavior, metabolism, and body energy balance.^14^ Adiponectin, a collagen-like protein expressed in adipose tissue,^29^ was found to play a markedly protective role in the pathogenesis of obesity-related disorders.^30^ A recent study conducted by Phillips et al reported that MHO individuals had leptin and adiponectin levels that were intermediate between those in MUO and metabolically healthy non-obesity adult.^31^ Our findings further confirmed that MHO children and adolescents also had L:A ratios that were intermediate between MUO and metabolically healthy normal-weight individuals. Thus, the L:A ratio might emerge as a significant discriminator between the MHO and MUO phenotypes among children and adolescents.

A recent study suggested that the L:A ratio deserves further consideration as a possible component of MS in Chinese children and adolescents.^16^ However, it is unclear whether adipokines can be used as additional components of MS to effectively explain the relationship between the MHO phenotype and CVD. Seth et al reported that increased circulating leptin levels are common in obesity and are independently associated with CVD in humans.^32^ Previous studies have demonstrated that hypoadiponectinemia is a predictor for the development of hypertension and a risk factor for atherosclerosis.^33^^,^^34^ In this study, our results also revealed a significant relationship between the adipokines levels and SBP as well as DBP. Moreover, the abnormal adipokines, including high leptin levels and high L:A ratio, showed an increased risk of developing hypertension when compared with individuals with normal adipokines.

Du et al reported that, in addition to MS, elevated levels of nontraditional risk factors and the visceral adiposity index scores in MHO individuals could contribute to the increased CVD risk observed in the long-term studies.^12^ In the present study, we found that, compared to metabolic healthy normal-weight individuals, the individuals with MHO phenotypes have a higher prevalence of low adiponectin and high leptin levels as well as a high L:A ratio, which play a role in increased risk of hypertension in MHO individuals. When hypertension outcomes were assessed in relation to baseline status, individuals with low adiponectin levels, high leptin levels, or a high L:A ratio experienced a non-significantly elevated risk of developing hypertension during the 6-year follow-up period compared with metabolically healthy normal-weight individuals. However, the MHO individuals with high leptin or a high L:A ratio were significantly more likely to developing hypertension when compared with metabolically healthy normal-weight individuals with normal adipokine levels.

The optimal BMI cut-off for overweight and obesity in Western population could be different to that of Asian subjects, because Asian children have different body fat distribution for the same level of BMI compared with African-American and Caucasian children.^35^ Therefore, the BMI cutoffs recommended by the Working Group on Obesity in China^36^ were also used to analyze the data (eTable 2, eTable 3, and eFigure 1). However, the results were similar with those overweight and obesity classified by BMI cutoffs recommended by the International Obesity Task Force (IOTF) reference.

There are several limitations to this study. First, because some baseline risk factor levels were different between those who did and those who did not attend follow-up, bias due to differential loss to follow-up is likely. Second, because the study is comprised of only Chinese children and adolescents, the results may not apply to other racial/ethnic populations. Third, other CVD risk factors, such as apolipoprotein and inflammatory markers, which were increased in MHO individuals, were not considered. Last, during the follow-up period, children became adolescents or young adult, so their hormones may have caused transient or physiological insulin resistance, leading to the changes in blood pressure and occasional hypertension.

Thus, further longitudinal studies on whether metabolically healthy obese children and adolescents still develop hypertension are required. Despite the limitations, this study is the first to introduce the relationship between the adipokines and hypertension in MHO individuals among Chinese children and adolescents. The results in the present study provide additional impetus for clinical studies and research for the potential existence of non-traditional risk factors in MHO individuals.

In conclusion, the present study has demonstrated that a considerable proportion of MHO individuals possess low adiponectin and high leptin levels, as well as high L:A ratio levels. The abnormal adipokine levels in the MHO adolescents and young adults contribute to the increased hypertension risk in these individuals. Therefore, the non-traditional risk factors, which are strongly predictive of the risk of future morbidity from hypertension, should be highlighted in MHO individuals among children and adolescents. In addition, caution is warranted in using the term “metabolically healthy” in future research that investigates the association between obesity and CVD outcomes.

## APPENDIX A. SUPPLEMENTARY DATA

eTable 1. Characteristics of followed and non-followed subjects at baseline

eTable 2. Relative risks for incident hypertension in relation to baseline leptin, adiponectin, and leptin:adiponectin ratio

eTable 3. Relative risks for incident hypertension according to different combinations of MHO phenotypes and abnormal adipokines

eFigure 1. Prevalence of abnormal adipokines among subjects by BMI and metabolic status at baseline. Abnormal adipokines were defined as adipokines levels ≥85th percentile for age and sex, with the exception of low adiponectin, which was defined as adiponectin levels ≤15th percentile for age and sex. Metabolic abnormalities were defined by the presence of two or more of the five components of MS. Obesity was defined based on Working Group on Obesity in China (WGOC) BMI cutoffs. The prevalence of high leptin, lower adiponectin and high leptin/adiponectin ratio in MHO individuals were significantly higher than in metabolically healthy normal-weight individuals (all P < 0.01). MHO, metabolically healthy obesity.
