# Supplementary material for: Adipokines are Associated With Hypertension in Metabolically Healthy Obese (MHO) Children and Adolescents: A Prospective Population-Based Cohort Study
Source: J Epidemiol. 2018 Jan 5;28(1):19–26. doi: 10.2188/jea.JE20160141 (PMC5742375; doi:10.2188/jea.JE20160141)
Supplement: Supplementary file 1 [file je-28-019-s001.pdf]

eTable 1. Characteristics of followed and non-followed subjects at baseline

Characteristics	Followed	Non-followed	t/χ^2	P-value
n	1,184	1,005		
Age, years	10.0 (2.1)	10.0 (2.1)	0.36	NS
Sex, boys, n (%)	643 (54.3)	561(55.8)	0.50	NS
Pubertal stage, prepuberty , n (%)[a]	292 (30.0)	203(17.6)	45.26	<0.001
BMI, kg/m^2	20.8 (4.5)	20.2 (4.6)	3.58	<0.001
WC, cm	68.8 (12.1)	67.2 (12.1)	3.07	0.002
FMP, %	23.4 (8.4)	21.6 (8.7)	5.47	<0.001
SBP, mm Hg	102 (10)	100 (11)	4.66	<0.001
DBP, mm Hg	64 (9)	65 (8)	4.26	<0.001
TG, mmol/L[b]	0.92 (0.68,1.24)	0.87(0.64,1.21)	1.62	NS
HDL-C, mmol/L	1.47 (0.32)	1.50 (0.33)	2.18	0.029
LDL-C, mmol/L	2.41 (0.67)	2.42 (0.68)	0.48	NS
TC, mmol/L	4.14 (0.74)	4.15 (0.73)	0.51	NS
Fathers' BMI	22.9 (7.87)	22.5 (5.97)	1.473	NS
Mother's BMI	20.0 (6.67)	19.3 (5.35)	2.735	0.006
Family history of hypertension, %[c]	12.9	11.9	0.181	NS
Annual family income, ten thousand Yuan [b]	3.6(1.8,6.0)	3.0(1.2,7.0)	1.109	NS

BMI, body mass index; DBP, diastolic blood pressure; FMP, fat mass percentage; HDL-C, high-density lipoprotein cholesterol; LDL-C, low-density lipoprotein cholesterol; MHO, metabolically healthy obesity; NS, not significant; SBP, systolic blood pressure; TC, total cholesterol; TG, triglyceride; WC, waist circumference.

Normally distributed data are expressed as mean (standard deviation). The median and interquartile range was used for skewed variables.

[a] Prepuberty: Tanner stage 1

[b] Skewed distributions were logarithmically transformed for statistical tests

[c] Presence of at least one parent with hypertension

eTable 2. Relative risks for incident hypertension in relation to baseline leptin, adiponectin, and leptin:adiponectin ratio

**Adipokines**	**Number of hypertension cases /Number of at risk**	**Model 1**		**Model 2**	
		**RR**	**95% CI**	**RR**	**95% CI**
Leptin, ug l^{-1}					
Normal	99/524	1(reference)		1(reference)	
High	35/77	3.57	2.17-5.89	2.10	1.19-3.73
Adiponectin, mg l^{-1}					
Normal	141/700	1(reference)		1(reference)	
Low	36/125	1.61	1.04-2.46	0.96	0.52-1.82
High leptin/adiponectin ratio					
Normal	98/519	1(reference)		1(reference)	
High	35/79	3.42	2.08-5.61	2.19	1.23-3.90

CI, confidence interval; RR, relative risk.

Model 1: unadjusted for confounding factors.

Model 2: Adjusted for age, sex, BMI, fathers' BMI, mothers' BMI, pubertal stage, physical activity and family history of hypertension.

eTable 3. Relative risks for incident hypertension according to different combinations of MHO phenotypes and abnormal adipokines

	Adipokines	Number of hypertension cases /Number of at risk	Model 1		Model 2	
			RR	95% CI	RR	95% CI
MHO	Low adiponectin					
-	-	29/323	1(reference)		1(reference)	
-	+	2/24	0.92	0.21-4.12	0.56	0.07-4.53
+	-	52/135	6.35	3.79-10.64	3.37	0.99-11.47
+	+	7/25	3.94	1.52-10.22	2.53	0.44-14.56
MHO	High leptin					
-	-	23/249	1(reference)		1(reference)	
-	+	0/1	-		-	
+	-	39/107	5.64	3.15-10.08	3.67	1.08-12.51
+	+	14/32	7.64	3.37-17.35	5.54	1.19-25.76
MHO	High leptin/adiponectin ratio					
-	-	22/244	1(reference)		1(reference)	
-	+	1/6	2.02	0.23-18.06	3.55	0.35-36.42
+	-	42/113	5.97	3.34-10.67	4.22	1.22-14.57
+	+	11/24	8.53	3.42-21.32	6.77	1.32-34.73

CI, confidence interval; MHO, metabolically healthy obesity; RR, relative risk.

Model 1: unadjusted for confounding factors.

Model 2: Adjusted for age, sex, BMI, fathers' BMI, mothers' BMI, pubertal stage, physical activity and family history of hypertension.

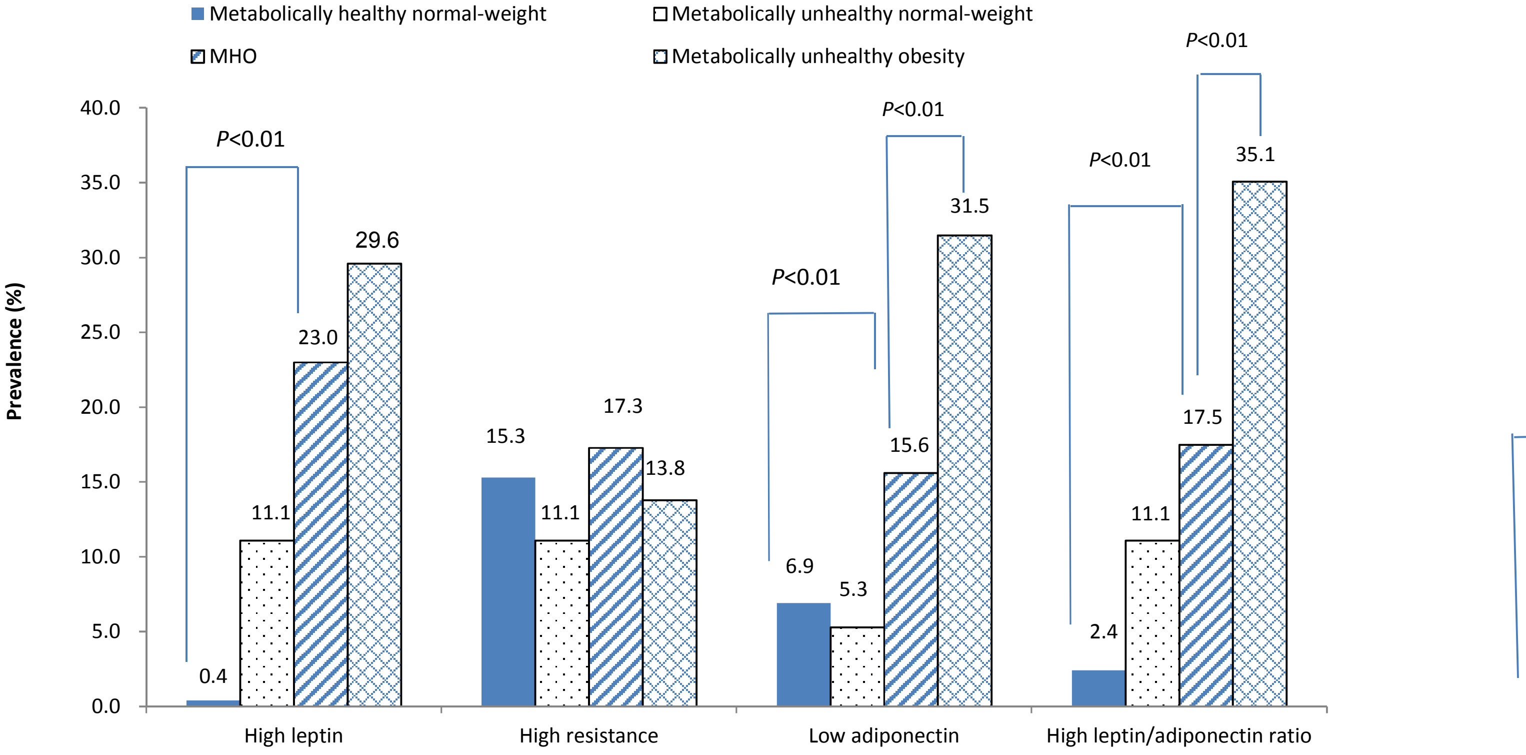

Figure1. Prevalence of abnormal adipokines among subjects by BMI and metabolic status at baseline. Abnormal adipokines were defined as adipokines levels ≥85th percentile for age and sex, with the exception of low adiponectin, which was defined as adiponectin levels ≤15th percentile for age and sex. Metabolic abnormalities were defined by the presence of two or more of the five components of MS. Obesity was defined based on Working Group on Obesity in China (WGOC) BMI cutoffs. The prevalence of high leptin, lower adiponectin and high leptin/adiponectin ratio in MHO individuals were significantly higher than in metabolically healthy normal-weight individuals (all $P<0.01$). MHO, metabolically healthy obesity.
